# Phase 1, randomized, double-blind, placebo-controlled, ascending single- and multiple-dose study of the safety, tolerability, and pharmacokinetics of intravenous xeruborbactam (QPX7728) in healthy adult subjects

**DOI:** 10.1128/aac.00784-25

**Published:** 2025-09-15

**Authors:** María Patricia Hernández-Mitre, Steven C. Wallis, Jeffery S. Loutit, David C. Griffith, Jason A. Roberts

**Affiliations:** 1 UQ Centre for Clinical Research, Faculty of Health, Medicine, and Behavioural Sciences (HMBS), The University of Queensland 1974, Brisbane, Queensland, Australia; 2 Qpex Biopharma, Inc 656306 , San Diego, California, USA; 3 Herston Infectious Diseases Institute (HeIDI), Metro North Health, Brisbane, Queensland, Australia; 4 Department of Intensive Care Medicine, Royal Brisbane and Women’s Hospital, Brisbane, Queensland, Australia; 5 Department of Pharmacy, Royal Brisbane and Women's Hospital 3883, Brisbane, Queensland, Australia; 6 UR UM 103, Division of Anesthesia Critical Care and Emergency and Pain Medicine, University of Montpellier, Nimes University Hospital, Nimes, France; Providence Portland Medical Center, Portland, Oregon, USA

**Keywords:** QPX7728, xeruborbactam, pharmacokinetics, safety, tolerability, Phase 1

## Abstract

The objective of this First-in-Human Phase 1, double-blind, randomized, placebo-controlled study was to assess the pharmacokinetics (PK), safety, and tolerability of the dual targeting beta-lactamase inhibitor xeruborbactam (QPX7728) in healthy adults. The study included single-ascending (SAD) and multiple-ascending dose (MAD) cohorts. Subjects received intravenous xeruborbactam at doses ranging from 250 to 2,000 mg. PK parameters of total and unbound xeruborbactam were derived from plasma, ultrafiltrate, and urine samples. Adverse events (AEs) were monitored and assessed throughout the study. Fifty-two subjects participated (39 xeruborbactam and 13 placebo). Following single doses, total and unbound xeruborbactam exhibited dose-dependent increases in exposure parameters (*C*_max_, AUC_0–24_, and AUC_0–INF_). Mean terminal half-life ranged from 26.8 to 32.0 h for total xeruborbactam, and from 20.7 to 22.4 h for unbound xeruborbactam. The mean fraction of the administered dose excreted in the urine ranged from 82.9% to 85.0% after single doses, and from 71.5% to 81.2% after multiple doses. After repeated 8-hourly dosing in MAD cohorts 7 and 8, the mean accumulation ratios for total xeruborbactam were 5.0 and 6.1. Plasma protein binding showed a concentration-dependent trend, with increased unbound xeruborbactam concentrations at higher doses. AEs were not different between xeruborbactam and placebo participants and included headache, vascular access site bruising and pain, and self-resolving mild increases in alanine transaminases. No severe or serious AEs were observed. Xeruborbactam was primarily eliminated renally. Dose proportionality was only observed for unbound xeruborbactam. Significant accumulation was noted with 8-hourly dosing. Xeruborbactam at doses up to 1,000 mg daily for 7–10 days was well tolerated, with no serious or life-threatening AEs. Xeruborbactam was primarily eliminated renally. Dose proportionality was only observed for unbound xeruborbactam. Significant accumulation was noted with 8-hourly dosing. Xeruborbactam at doses up to 1,000 mg daily for 7–10 days was well tolerated, with no serious or life-threatening AEs.

## INTRODUCTION

The Centers for Disease Control and Prevention has listed carbapenem-resistant *Enterobacteriaceae* and *Acinetobacter* as urgent threats, and multidrug-resistant *Pseudomonas aeruginosa* and extended-spectrum beta-lactamase (ESBL)-producing *Enterobacteriaceae* as serious threats (1). Consistent with the global nature of these resistant bacteria, the World Health Organization has designated carbapenem-resistant ESBL-producing *Enterobacteriaceae*, carbapenem-resistant *Acinetobacter baumannii*, and carbapenem-resistant *P. aeruginosa* as critical needs for new drug therapy (2).

Qpex Biopharma, Inc. (Qpex) is developing xeruborbactam to partner with approved beta-lactam antibiotics, as oral and intravenous (IV) therapy for treating serious infections due to gram-negative pathogens. Xeruborbactam is a dual-targeting beta-lactamase inhibitor, with activity against both serine and metallo-beta-lactamases, including class A (ESBLs, KPC, and PER), class B (NDM and VIM), class C (AmpC), and class D (OXAs) that can hydrolyze all different classes of beta-lactams and can be found in Enterobacterales, *A. baumannii*, and *P. aeruginosa*.

This First-in-Human Phase 1 study assessed the pharmacokinetics (PK), safety, and tolerability of single- and multiple-ascending doses (SAD and MAD) of IV xeruborbactam in healthy adults.

## RESULTS

Fifty-two subjects were included in the study: 39 subjects received xeruborbactam and 13 received placebo. One subject from the MAD cohort 8 (500 mg, then 250 mg 8-hourly) withdrew from the trial and was consequently excluded from total and unbound xeruborbactam and urine analyzes. Another subject from the MAD cohort 7 (1,000 mg, then 500 mg 8-hourly) had no urine sample concentration at 8–12 h on Day 12 due to a recorded volume of 0 mL, and a subject from the SAD cohort 2 (500 mg) had an unquantified urine sample at 0–4 h. The demographics for all subjects are summarized in Table 1. Overall, demographics were broadly consistent between the pooled xeruborbactam and placebo groups.

**TABLE 1:** Demographics and baseline characteristics (mean ± SD) of the single- and multiple-dose cohorts

Single-ascending dose cohorts							
	Xeruborbactam dose						
Demographics	250 mg	500 mg	1,000 mg	2,000 mg	Pooled xeruborbactam	Pooled placebo	Overall
*N*	5	4	6	6	21	7	28
Age (years)	26 (±6.1)	24.3 (±7.2)	29.8 (±13.8)	25 (±6.5)	26.5 (±8.9)	27.7 (±10.4)	26.8 (±9.1)
Male, *n* (%)	5 (100%)	4 (100%)	5 (83.3%)	6 (100%)	20 (95.2%)	7 (100%)	27 (96.4%)
Race, *n* (%)							
Asian	1 (20%)	1 (25%)	1 (16.7%)	3 (50%)	6 (28.6%)	2 (28.6%)	8 (28.6%)
White	4 (80%)	3 (75%)	5 (83.3%)	3 (50%)	15 (71.4%)	5 (71.4%)	20 (71.4%)
Not Hispanic or Latino, *n* (%)	4 (80%)	4 (100%)	6 (100%)	5 (83.3%)	19 (90.5%)	7 (100%)	26 (92.9%)
Height (cm)^*a*^	173.9 (±2.7)	176.6 (±7.2)	173.8 (±10.6)	176.2 (±8.3)	175.0 (±7.5)	178.6 (±9.4)	175.9 (±8.0)
Weight (kg)^*a*^	72.2 (±11.8)	73.0 (±6.6)	76.5 (±9.7)	71.5 (±7.0)	73.4 (±8.6)	84.2 (±10.5)	76.1 (±10.1)
BMI (kg/m^2^)^*a*^	23.9 (±4.2)	23.7 (±3.6)	25.4 (±2.9)	23.3 (±1.8)	24.1 (±3.0)	26.4 (±1.6)	24.7 (±2.9)

Multiple-ascending dose cohorts						
	Xeruborbactam dose					
Demographics	1,000 mg LD/500 mg q8h	500 mg LD/250 mg q8h	1,000 mg QD	Pooled xeruborbactam	Pooled placebo	Overall
*N*	6	6	6	18	6	24
Age (years)	30.5 (±12.2)	32.5 (±9.9)	33.8 (±8.0)	32.3 (±9.7)	38.2 (±11.3)	33.8 (±10.2)
Male, *n* (%)	5 (83.3%)	5 (83.3%)	6 (100%)	16 (88.9%)	6 (100%)	22 (91.7%)
Race, *n* (%)						
Asian	1 (16.7%)	0	2 (33.3%)	3 (16.7%)	1 (16.7%)	4 (16.7%)
Black or African	0	1 (16.7%)	1 (16.7%)	2 (11.1%)	0	2 (8.3%)
American						
White	4 (66.7%)	5 (83.3%)	3 (50%)	12 (66.7%)	4 (66.7%)	16 (66.7%)
Other	1 (16.7%)	0	0	1 (5.6%)	1 (16.7%)	2 (8.3%)
Not Hispanic or Latino, *n* (%)	6 (100%)	6 (100%)	5 (83.3%)	17 (94.4%)	5 (83.3%)	22 (91.7%)
Height (cm)^*a*^	172.4 (±9.9)	176.6 (±7.4)	173.5 (±10.4)	174.2 (±8.9)	172.1 (±1.5)	173.7 (±7.8)
Weight (kg)^*a*^	75.4 (±12.9)	77.2 (±13.0)	78.1 (±11.3)	76.9 (±11.7)	75.9 (±6.9)	76.7 (±10.6)
BMI (kg/m^2^)^*a*^	25.2 (±4.1)	24.9 (±3.05)	25.9 (±2.2)	25.3 (±3.0)	25.8 (±2.6)	25.5 (±2.9)

^*a*^ At screening; BMI, body mass index; LD, loading dose; q8h, 8-hourly; QD, once-daily.

### PK of total and unbound xeruborbactam following single doses

**Fig 1:**
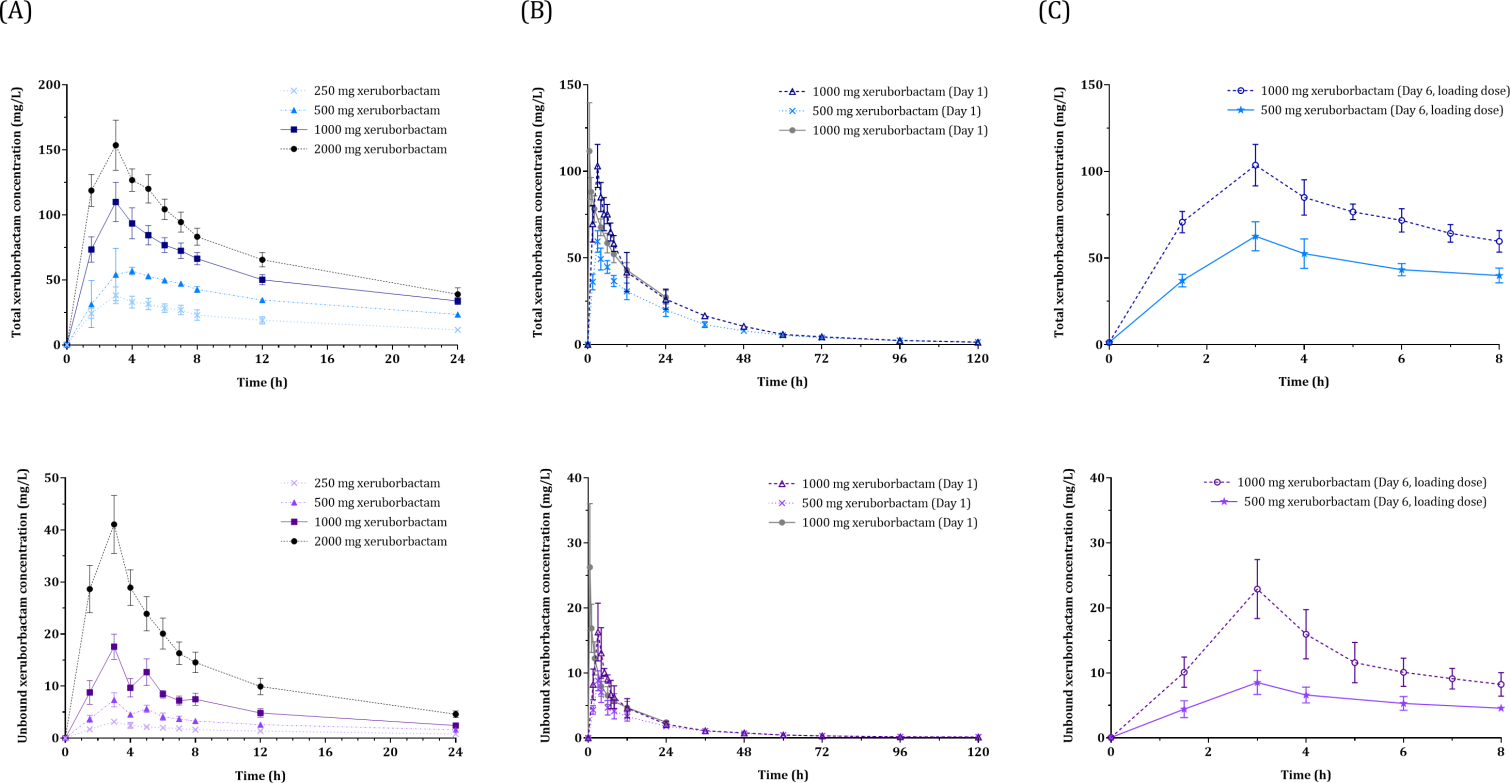
Total and unbound xeruborbactam concentrations (mean ± SD) for SAD cohorts (**A**), and MAD cohorts on Day 1 (**B**) and Day 6 (**C**).

Following a single-dose administration, total and unbound xeruborbactam concentrations were measured for up to 24 h across all SAD cohorts and up to 120 h in Day 1 MAD cohorts (Fig. 1). The total and unbound xeruborbactam exposure parameters—maximum observed plasma concentration (*C*_max_), area under the curve from 0 to 24 h (AUC_0–24_), and AUC from 0 extrapolated to ∞ (AUC_0–INF_)—exhibited a dose-dependent increase. The mean terminal half-life (*t*_1/2_) ranged from 14.7 to 18.6 h for total xeruborbactam and 9.7 to 15.3 h for unbound xeruborbactam across the SAD cohorts due to sampling limited to 24 h. Furthermore, mean volume of distribution (Vz) and total body clearance (CL) values varied from 8.0 to 16.4 L and from 0.34 to 0.78 L/h for total, and from 73.0 to 110.1 L and from 4.8 to 5.7 L/h for unbound xeruborbactam, respectively (Table 2).

**TABLE 2:** Xeruborbactam total and unbound pharmacokinetic parameters (mean ± SD) by cohort following single doses in the single-ascending dose (SAD) cohorts, and on Days 1 and 6 in the multiple-ascending dose (MAD) cohorts^*a*^^,^^*d*^

	SAD cohorts				MAD cohorts				
					Day 1			Day 6 (loading dose)	
Xeruborbactam dose	250 mg	500 mg	1,000 mg	2,000 mg	1,000 mg	500 mg	1,000 mg QD	1,000 mg	500 mg
*N*	5	4	6	6	6	5	6	6	5
Infusion length (h)	3	3	3	3	3	3	0.5	3	3
Total xeruborbactam									
*C*_max_ (mg/L)	38.16 ± 6.32	62.25 ± 3.94	110.88 ± 15.13	153.50 ± 19.23	103.12 ± 12.38	59.50 ± 6.22	111.68 ± 27.90	103.62 ± 11.99	62.58 ± 8.38
*T*_max_ (h)	3.00 ± 0.00	3.25 ± 0.50	3.17 ± 0.41	3.00 ± 0.00	3.00 ± 0.00	3.00 ± 0.00	0.50 ± 0.00	3.00 ± 0.00	3.00 ± 0.00
AUC_0–t_ (mg × h/L)^*b*^	477. 42 ± 65.34	838.47 ± 33.41	1,334.56 ± 87.57	1,763.53 ± 140.82	1,849.39 ± 236.72	1,311.18 ± 134.78	1,147.81 ± 112.68	563.56 ± 45.56	339.46 ± 34.03
AUC_0–INF_ (mg × h/L)	746.33 ± 88.26	1,459.83 ± 152.46	2,094.61 ± 175.19	2,589.50 ± 302.13	1,901.32 ± 239.12	1,376.54 ± 157.54	1,833.18 ± 254.38	1,276.25 ± 347.34	1,208.41 ± 792.05
*t*_1/2_ (h)	16.31 ± 1.99	18.59 ± 2.66	16.03 ± 2.62	14.72 ± 1.94	26.77 ± 5.80	31.97 ± 3.66	17.31 ± 2.22	8.19 ± 3.25	14.61 ± 11.43
Vz (L)	8.01 ± 1.69	9.16 ± 0.38	11.02 ± 1.32	16.43 ± 1.47	20.65 ± 5.39	16.80 ± 1.51	13.68 ± 1.25	9.05 ± 1.47	8.28 ± 1.51
CL (L/h)	0.34 ± 0.05	0.35 ± 0.03	0.48 ± 0.04	0.78 ± 0.09	0.53 ± 0.08	0.37 ± 0.04	0.56 ± 0.08	0.83 ± 0.20	0.52 ± 0.20
Unbound xeruborbactam									
*ub*C_max_ (mg/L)	3.13 ± 0.31	7.35 ± 1.36	17.55 ± 2.41	41.07 ± 5.58	16.68 ± 3.98	8.80 ± 1.54	26.27 ± 9.78	22.90 ± 4.55	8.53 ± 1.86
*ub*T_max_ (h)	3.00 ± 0.00	3.00 ± 0.00	3.00 ± 0.00	3.00 ± 0.00	3.17 ± 0.41	3.00 ± 0.00	0.50 ± 0.00	3.00 ± 0.00	3.00 ± 0.00
*ub*AUC_0–t_ (mg × h/L)^*c*^	34.05 ± 5.99	70.22 ± 5.10	142.07 ± 14.93	323.78 ± 42.73	181.49 ± 25.79	131.59 ± 24.39	139.23 ± 27.52	94.28 ± 18.45	42.26 ± 8.11
*ub*AUC_0–INF_ (mg × h/L)	51.30 ± 10.10	104.52 ± 14.15	177.94 ± 18.69	387.39 ± 52.68	184.89 ± 26.38	136.17 ± 25.48	184.33 ± 32.39	169.88 ± 33.64	109.53 ± 46.43
*ub*t_1/2_ (h)	15.28 ± 2.00	15.06 ± 2.27	10.48 ± 1.58	9.68 ± 1.08	20.66 ± 5.23	22.42 ± 7.42	13.05 ± 1.91	6.67 ± 3.30	10.49 ± 8.15
*ub*Vz (L)	110.08 ± 22.24	103.90 ± 6.84	85.31 ± 13.49	72.95 ± 10.78	163.06 ± 47.14	120.91 ± 41.03	104.93 ± 24.37	55.93 ± 21.16	63.76 ± 20.26
*ub*CL (L/h)	5.02 ± 0.96	4.84 ± 0.58	5.67 ± 0.55	5.25 ± 0.74	5.49 ± 0.72	3.78 ± 0.75	5.56 ± 0.91	6.14 ± 1.52	5.08 ± 1.56
Xeruborbactam in urine									
Ae_0–120_ (mg)	–^*e*^	–	–	–	828.93 ± 112.85	425.05 ± 34.49	–	–	–
Fe (%)	–	–	–	–	82.89 ± 11.28	85.01 ± 6.90	–	–	–
CL_R_ (L/h)	–	–	–	–	0.44 ± 0.04	0.31 ± 0.03	–	–	–

^*a*^ QD, once daily.

^*b*^ AUC_0–8_ for Day 6 MAD cohorts; AUC_0–24_ for SAD cohorts and Day 1 MAD 1,000 mg QD dose; AUC_0–120_ for Day 1 MAD 1,000 and 500 mg doses.

^*c*^ *ub*AUC_0–8_ for Day 6 MAD cohorts; *ub*AUC_0–24_ for SAD cohorts and Day 1 MAD 1,000 mg QD dose; *ub*AUC_0–120_ for Day 1 MAD 1000 and 500 mg doses.

^*d*^ Urine pharmacokinetics are presented only for Day 1 MAD 1,000 and 500 mg doses.

^*e*^ –, not applicable.

### PK of xeruborbactam in urine following single doses

The amount of xeruborbactam excreted in urine (Ae) over 24 h ranged from 110.7 mg for the 250 mg dose to 1,103.0 mg for the 2,000 mg dose, with the mean fraction of the administered dose excreted in the urine (fe) varying from 40.4% to 56.6% due to limited sampling. For Day 1 MAD cohorts, xeruborbactam exhibited a mean Ae over 120 h of 425.1 mg for the 500 mg dose and 828.9 mg for the 1,000 mg dose, with mean fe ranging from 82.9% to 85.0%. The mean renal clearance (CL_R_) across all SAD and Day 1 MAD cohorts ranged from 0.14 to 0.44 L/h (Table 2).

### PK of total and unbound xeruborbactam following multiple doses

**Fig 2:**
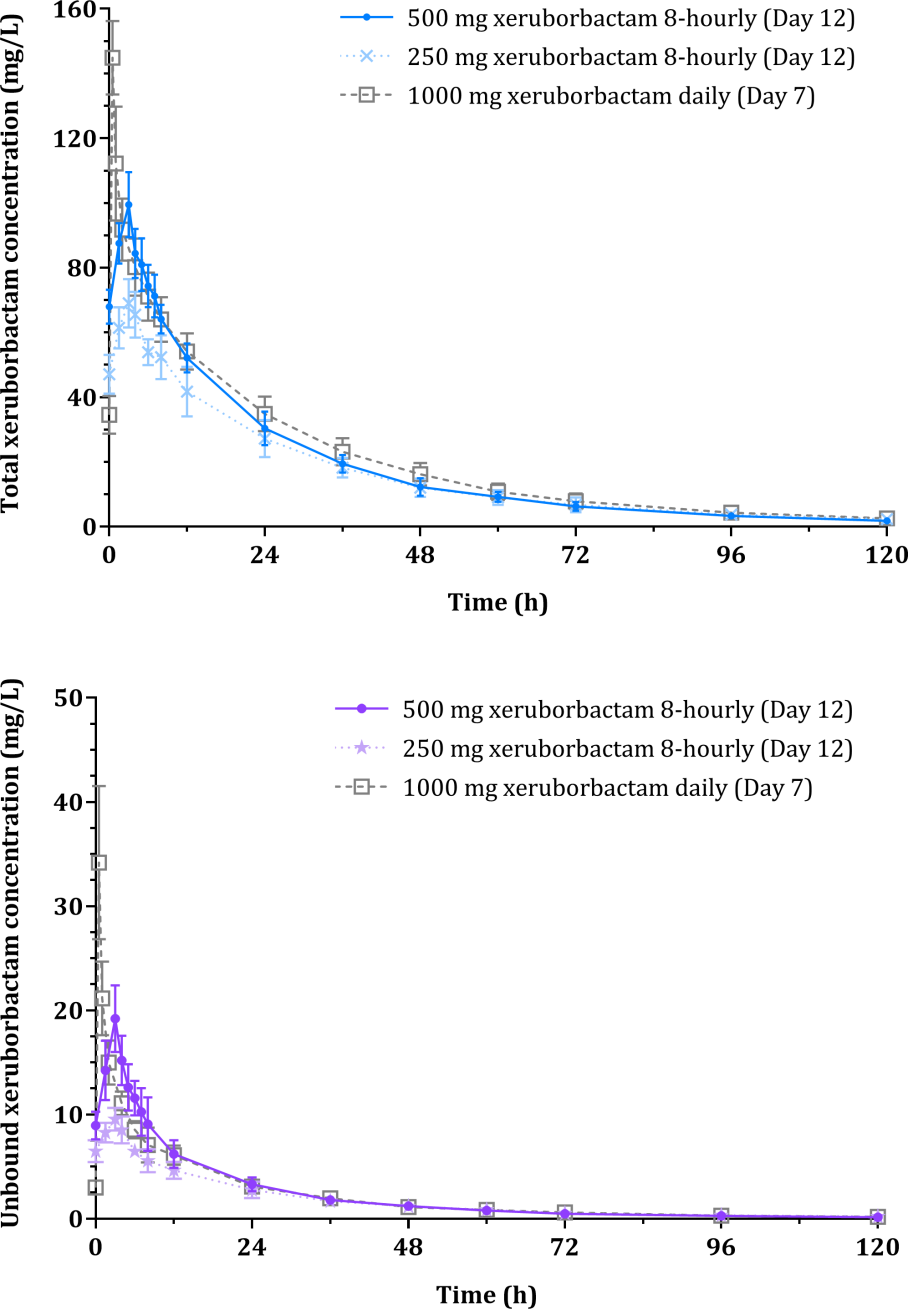
Total and unbound xeruborbactam concentrations (mean ± SD) following multiple dosing in the MAD cohorts.

Figure 2 illustrates the mean PK profiles of total and unbound xeruborbactam following multiple dose administration of 500 and 250 mg xeruborbactam 8-hourly, and 1,000 mg once-daily. Following multiple doses of xeruborbactam, a dose-dependent increase in total and unbound xeruborbactam exposure parameters was observed. The mean *t*_1/2_ ranged from 24.9 to 31.0 h for total xeruborbactam and from 25.3 to 27.9 h for unbound xeruborbactam. Mean Vz and CL values varied from 23.8 to 29.9 L and from 0.53 to 0.77 L/h for total, and from 167.6 to 203.8 L and from 4.2 to 5.6 L/h for unbound xeruborbactam, respectively (Table 3).

**TABLE 3:** Xeruborbactam total, unbound, and urine pharmacokinetic parameters (mean ± SD) following multiple doses in the multiple-ascending dose (MAD) cohorts^*a*^

	MAD cohorts		
	Day 12		Day 7
Xeruborbactam dose	500 mg q8h	250 mg q8h	1,000 mg QD
*N*	6	5	6
Infusion length (h)	3	3	0.5
Total xeruborbactam			
*C*_max_ (mg/L)	99.85 ± 9.74	69.00 ± 7.49	144.83 ± 11.36
*T*_max_ (h)	2.75 ± 0.61	3.00 ± 0.00	0.5 ± 0.00
AUC_0–*t*_ (mg × h/L)^*b*^	649.12 ± 44.27	471.60 ± 45.19	1,428.55 ± 153.25
AUC_0–120_ (mg × h/L)	2,234.66 ± 251.93	1,926.73 ± 341.36	2,498.84 ± 367.18
AUC_0–INF_ (mg*h/L)	2,296.78 ± 265.76	2,029.39 ± 379.60	2,607.52 ± 402.09
*t*_1/2_ (h)	24.91 ± 1.78	30.98 ± 1.47	29.31 ± 2.12
Vz (L)	27.86 ± 3.64	23.80 ± 1.53	29.92 ± 4.22
CL (L/h)	0.77 ± 0.06	0.53 ± 0.06	0.71 ± 0.08
Unbound xeruborbactam			
*ub*C_max_ (mg/L)	19.20 ± 3.21	9.56 ± 1.11	34.17 ± 7.36
*ub*T_max_ (h)	3.00 ± 0.00	3.20 ± 0.45	0.50 ± 0.00
*ub*AUC_0–*t*_ (mg × h/L)^*c*^	105.99 ± 17.34	60.27 ± 6.80	180.74 ± 23.73
*ub*AUC_0–120_ (mg × h/L)	270.90 ± 49.61	202.16 ± 42.20	263.79 ± 41.37
*ub*AUC_0–INF_ (mg × h/L)	276.17 ± 49.81	209.79 ± 42.85	269.82 ± 43.82
*ub*t_1/2_ (h)	26.56 ± 4.26	27.94 ± 12.24	25.33 ± 1.89
*ub*Vz (L)	185.96 ± 48.47	167.64 ± 72.30	203.75 ± 16.19
*ub*CL (L/h)	4.82 ± 0.76	4.19 ± 0.48	5.62 ± 0.76
Xeruborbactam in urine			
Ae_0–*t*_ (mg)^*d*^	405.82 ± 47.70	194.11 ± 56.83	715.29 ± 140.69
Fe (%)	81.16 ± 9.54	77.64 ± 22.73	71.53 ± 14.07
CL_R_ (L/h)	0.63 ± 0.08	0.41 ± 0.12	0.51 ± 0.12

^*a*^ q8h, every 8 h; QD, once daily.

^*b*^ AUC_0–8_ for Day 12 MAD 500 mg q8h and 250 mg q8h doses; AUC_0–24_ for Day 7 MAD 1,000 mg QD dose.

^*c*^ *ub*AUC_0–8_ for Day 12 MAD 500 mg q8h and 250 mg q8h doses; *ub*AUC_0–24_ for Day 7 MAD 1,000 mg QD dose.

^*d*^ Ae_0–8_ for Day 12 MAD 500 mg q8h and 250 mg q8h doses; Ae_0–24_ for Day 7 MAD 1,000 mg QD dose.

### PK of xeruborbactam in urine following multiple doses

Xeruborbactam mean Ae over 8 h ranged from 194.1 mg for the 250 mg dose to 405.8 mg for the 500 mg dose. The mean Ae over 24 h was 715.3 mg for the 1,000 mg dose. The mean fe across cohorts varied from 71.5% to 81.2%, while the mean CL_R_ ranged from 0.41 to 0.63 L/h (Table 3).

### Dose proportionality

Linear regression analysis of the SAD cohorts provided evidence of proportionality for total (*y* = 0.7024 *x* + 445.01; *r* = 0.9653) and unbound (*y* = 0.1661 *x* – 13.256; *r* = 0.9983) xeruborbactam, as AUC_0–24_ increased with escalating doses. The slopes were statistically greater than 0 (*P* = 0.035 and *P* = 0.002 for total and unbound xeruborbactam, respectively). Analysis of variance (ANOVA) revealed statistically significant differences in the total xeruborbactam exposure parameters among cohorts (*P* < 0.001), confirmed by a Tukey-Kramer Multiple-Comparison post hoc test. Dose proportionality for unbound xeruborbactam was demonstrated by ANOVA, as no differences were found for AUC_0–24_ and AUC_0–INF_. However, a statistically significant difference (*P* < 0.001) was found for *C*_max_. The subsequent Tukey-Kramer Multiple-Comparison post hoc test confirmed a difference in the mean normalized *C*_max_ between the 250 and 2,000 mg cohorts.

### Accumulation

The mean ± SD accumulation ratios of xeruborbactam following repeated 8-hourly administrations were 5.01 ± 0.32 and 6.10 ± 0.26 for the 500 and 250 mg 8-hourly regimens, respectively. The mean accumulation ratio over 7 days of once-daily dosing was 1.25 ± 0.09.

### Protein binding

Xeruborbactam exhibited concentration-dependent plasma protein binding (Fig. S1). The individual plasma protein binding values ranged from 66.5% to 97.0%, with a mean plasma protein binding of 88.0%.

### Safety and tolerability

In the SAD cohorts, 9/21 (42.9%) subjects in the xeruborbactam group and 3/7 (42.9%) subjects in the placebo group reported at least one treatment-emergent adverse event (TEAE). There was no evidence of increasing incidence or severity of TEAEs with increasing dose. The only TEAEs observed in more than one subject who received xeruborbactam were headache (2/21, 9.5%), contact dermatitis (2/21, 9.5%), and vascular access site pain (2/21, 9.5%). There were no serious TEAEs, severe TEAEs, or TEAEs leading to study discontinuation and no TEAEs leading to withdrawal of study drug.

In the MAD cohorts, 11/17 (64.7%) xeruborbactam-treated subjects and 6/6 (100%) placebo-treated subjects reported at least one TEAE. TEAEs observed in two or more subjects included transaminases increased (4/17, 23.5%), headache (2/17, 11.8%), and infusion site bruising (2/17, 11.8%). The most common TEAE reported in subjects who received xeruborbactam was increased plasma transaminases. Of the four subjects affected, one subject from the MAD cohort 8 (500 mg, then 250 mg 8-hourly) experienced a moderate alanine aminotransferase (ALT) elevation (>3 to <5 × above the upper limit of normal, ULN), while three subjects from cohort 7 (1,000 mg, then 500 mg 8-hourly) had ALT elevations—two mild (<3 × ULN) and one severe (>5 to <20 × ULN). Elevations were first observed on Days 5–10 of dosing, with peak values occurring 1–3 days after the final dose. Three of the four subjects were asymptomatic; one reported fatigue, which was considered unlikely to be related to treatment. ALT elevations resolved within 2–4 weeks of completion of dosing, did not lead to treatment withdrawal, and were not associated with bilirubin elevations. The majority of TEAEs reported were mild in severity. One subject experienced a severe TEAE, and two subjects experienced moderate TEAEs. The severe TEAE and one of the moderate TEAEs were increased plasma transaminases (as noted above), and the other moderate TEAE was a toothache. There were no serious TEAEs or TEAEs leading to study discontinuation or study drug withdrawal.

## DISCUSSION

In this study of the plasma and urine PK of xeruborbactam in healthy adult subjects, we observed dose-dependent increases in the total and unbound Cmax, AUC_0–24_, and AUC_0–INF_, in both SAD and MAD cohorts. In urine, the fraction excreted of xeruborbactam remained relatively consistent across single and multiple doses (78–85%). Overall, renal clearance was the primary route of elimination of xeruborbactam.

Graphical evaluation of the SAD cohorts suggested dose proportionality for the AUC_0–24_ of total xeruborbactam, but ANOVA of dose-normalized parameters indicated less than dose proportional increases in *C*_max_, AUC_0–24_, and AUC_0–INF_ across the 250–2,000 mg range. Conversely, unbound xeruborbactam exposure was dose proportional, except for *C*_max_, likely due to intrinsic metabolic and renal clearance rather than protein binding dynamics. The *C*_max_ exception may result from transient saturation of binding sites or altered absorption kinetics at higher doses, momentarily affecting peak unbound concentrations without significantly impacting overall exposure. Unlike total concentrations, unbound xeruborbactam is the pharmacologically active fraction subject to elimination. Saturable plasma protein binding, as observed with ceftriaxone (3), can cause a nonlinear increase in the unbound fraction at higher doses, deviating total drug exposure from dose proportionality while unbound drug clearance remains unchanged.

The potential for accumulation of xeruborbactam was assessed using different methods for the 8-hourly cohorts and once-daily MAD cohorts. Xeruborbactam accumulated with 8-hourly and once-daily administration, which is consistent with expectations given its half-life of around 30 h. For the 8-hourly regimens using 3 h infusions, a loading dose was used to achieve appropriate exposures rapidly, followed by maintenance dosing to sustain those exposures while taking advantage of the accumulation. Steady state was achieved by the fifth or sixth dose, and the regimen was designed accordingly to maintain unbound concentrations within the expected pharmacodynamic range. Higher accumulation was observed when xeruborbactam was administered 8-hourly compared to once daily.

The plasma protein binding of xeruborbactam across the studied dose range of 250 to 2,000 mg was concentration-dependent, with unbound concentrations increasing with rising total xeruborbactam concentrations. This finding directly relates to the observations where unbound xeruborbactam concentrations increase proportionately with dose, while the increase in total xeruborbactam concentrations is less than dose proportional. The saturable protein binding is pharmacologically evident but unlikely to be clinically significant (4).

The most common AE across the MAD cohorts was an ALT increase. The ALT elevations were often associated with aspartate aminotransferase (AST) elevations but were not associated with bilirubin elevations. No severe ALT elevations were observed in any subjects who received a dose of 1,000 mg or less per day of xeruborbactam, over 7–10 days of dosing. Only one subject from the MAD cohort 8 (500 mg, then 250 mg 8-hourly) had an ALT elevation greater than 3 × ULN. This subject had a baseline ALT of 41 U/L (ULN 51 U/L), which increased to a maximum of 156 U/L 3 days after the completion of 7 days of dosing and returned to normal within 4 weeks. The subject was asymptomatic, with the possible exception of a TEAE of fatigue that began on the same day as the TEAE of transaminase increase, but this was considered unlikely to be treatment-related. There was no increase in bilirubin above the ULN observed. Based on the increased incidence of AEs observed in the subjects who received more than 1,000 mg per day of QPX7728, future studies will investigate doses of QPX7728 at 1,000 mg per day or less.

Other than the above-mentioned transaminase increases, there were no clinically significant trends in other clinical laboratory parameters, and no significant changes in vital signs, physical examinations, or electrocardiogram parameters in the study.

### Conclusion

The PK of total and unbound xeruborbactam, along with the urine PK, was investigated in healthy adult subjects following single and multiple IV doses ranging from 250 to 2,000 mg. Renal clearance was the primary route of elimination of xeruborbactam. Dose proportionality was observed for unbound xeruborbactam in plasma, although it was not demonstrated across the studied dose range for total xeruborbactam in plasma due to saturable protein binding. Minor drug accumulation of xeruborbactam was observed during once-daily dosing for 7 days, whereas significant accumulation occurred during 8-hourly dosing for 7 days. A concentration-dependent plasma protein binding trend was observed. Overall, xeruborbactam at a daily dose of 1,000 mg or less for 7–10 days was well-tolerated. No SAEs or life-threatening AEs were observed. The most common AE was a mild increase in ALT, which was asymptomatic and was not associated with bilirubin elevations or other measures of liver function.

## MATERIALS AND METHODS

The study protocol was reviewed and approved on 27 July 2020 by the Bellberry Human Research Ethics Committee, which was appropriately constituted and operated in accordance with principles and requirements described per ICH guidelines.

### Study design

This was a double-blind, randomized, placebo-controlled, sequential ascending single- and multiple-dose study of IV xeruborbactam. In the SAD portion, subjects received a single IV dose of 250, 500, 1,000, or 2,000 mg of xeruborbactam or placebo as a 3-h infusion on Day 1 (cohorts 1, 2, 4, and 6). In the MAD portion, cohorts 7 and 8 received a single IV dose of 1,000 and 500 mg, respectively, on Day 1. This single dose on Day 1 allowed for complete PK characterization and safety monitoring prior to initiating multiple dosing. On Day 6, subjects received an IV loading dose (same as Day 1) or placebo followed by multiple doses of 500 and 250 mg, respectively, 8-hourly until Day 12. Doses were administered as a 3-h infusion. For the MAD cohort 9, subjects received 1000 mg of xeruborbactam or placebo as a 30 min infusion once daily, with the last dose on Day 7 (Supplementary material).

### Subjects

Healthy adult subjects aged 18–55 years, with body mass index (BMI) 18.5–29.9 kg/m^2^, and weighing between 55 and 100 kg, were enrolled. All participants provided written informed consent. Exclusion criteria and data collected are detailed in the Supplementary Material.

### Blood and urine sampling

Blood and urine samples were collected per the clinical protocols at the time points delineated in Table S1.

### Sample analysis

The concentration of xeruborbactam in plasma and urine samples was quantified using a validated high-performance liquid chromatography with tandem mass spectrometry (HPLC-MS/MS) method. Plasma samples, with K2EDTA as anticoagulant, used QPX7834 as internal standard, while urine samples used xeruborbactam-d_2_. Both types of samples were precipitated with a methanol:acetonitrile solution. The supernatant was further diluted, and 5 µL of each sample was injected into the HPLC-MS/MS system. HPLC separation was performed using a Waters Atlantis dC18 column, employing electrospray positive ionization and MS/MS mode.

The calibration range was from 0.2 to 100 mg/L. For xeruborbactam in plasma, the intra- and inter-day accuracy ranged from −4.3% to 6.3% and −3.3% to 4.3%, respectively, with precision between 2.0% and 3.5% and between 2.6% and 3.3%. In urine, the intra- and inter-day accuracy ranged from −6.3% to 0.3% and −4.2% to -1.7%, respectively, with precision between 1.1% and 2.5% and between 1.4% and 3.9%.

### Pharmacokinetics

Non-compartmental analysis techniques (Phoenix WinNonlin version 8.3.5.340, Certara) were used to derive total and unbound PK parameters from individual plasma concentrations, as well as urine PK parameters. Summary statistics (*N*, mean, and SD) were tabulated by cohort for each PK parameter. The mean maximum observed plasma concentration (*C*_max_), time of observed plasma concentration (*T*_max_), area under the curve from 0 to time *t* (AUC_0–*t*_), AUC from 0 extrapolated to ∞ (AUC_0–INF_), terminal half-life (*t*_1/2_), volume of distribution (Vz), and total body clearance (CL) were obtained for total and unbound xeruborbactam. Urine PK parameters included the amount of drug excreted in the urine from 0 to time *t* (Ae_0–*t*_), fraction of the administered dose excreted in the urine (fe), and renal clearance (CL_R_) (calculated as Ae_0–*t*_/AUC_0–*t*_ for single doses, and Ae_0–tau_/AUC_0–tau_ for multiple doses, corresponding to the dosing interval at steady state). All concentration values below the quantification limit were treated as missing.

### Dose proportionality

Dose proportionality was examined using total and unbound xeruborbactam AUC_0–24_ values from SAD cohorts through linear regression analysis performed in Microsoft Excel. The linear regression model was expressed as AUC = *β* × dose + *μ*, where *β* represents the slope of the regression model and *μ* is the intercept. If the slope was significantly greater than 0 (*P* < 0.05), then some evidence of proportionality was accepted.

Statistical assessment of dose proportionality used the total and unbound xeruborbactam PK exposure parameters Cmax, AUC_0–24_, and AUC_0–INF_. Each subject’s parameter values were normalized to 250 mg. ANOVA was conducted for normally distributed data, while the Kruskal-Wallis test was used for non-normally distributed data. Normality of data were assessed using the Shapiro-Wilk test. Statistical analyzes were performed with IBM SPSS Statistics version 28.0.1.0.

### Accumulation of total xeruborbactam in plasma exposure

The accumulation of total xeruborbactam after repeated administrations was assessed in MAD cohorts 7 and 8 using the dosing interval (*τ* = 8 h) and elimination rate constant (ke), calculated as 1/(1 − *e*^−*ke×τ*^). In the MAD cohort 9, accumulation from a single dose to steady state with repeated administrations was determined as the ratio of Day 7 AUC_0–24_/Day 1 AUC_0–24_.

### Protein binding

Plasma protein binding was calculated using plasma and ultrafiltrate data collected at the same time point. The formula used was ((total drug – unbound drug)/total drug) × 100, where the total drug represents the xeruborbactam concentration in plasma, and the unbound drug is the xeruborbactam concentration in ultrafiltrate.

### Safety and tolerability

Each subject was monitored for the occurrence of AEs, including serious AEs, for the duration of the study. An AE was defined as any unfavorable and unintended sign, symptom, or disease temporally associated with use of xeruborbactam. Each AE was assessed for its relationship to the drug treatment (not related, unlikely, possible, and probable) with signs or symptoms graded on a 5-point severity scale (mild, moderate, severe, life-threatening, and death). AEs were tabulated and summarized for description purposes only.

## SUPPLEMENTAL MATERIAL

Supplemental material aac.00784-25-s0001.docx Fig. S1; Table S1.

ASM does not own the copyrights to Supplemental Material that may be linked to, or accessed through, an article. The authors have granted ASM a non-exclusive, world-wide license to publish the Supplemental Material files. Please contact the corresponding author directly for reuse.
