# Supplementary material for: Phase 1, randomized, double-blind, placebo-controlled, ascending single- and multiple-dose study of the safety, tolerability, and pharmacokinetics of intravenous xeruborbactam (QPX7728) in healthy adult subjects
Source: Antimicrob Agents Chemother. 2025 Sep 15;69(10):e00784-25. doi: 10.1128/aac.00784-25 (PMC12486803; doi:10.1128/aac.00784-25)
Supplement: Supplemental material — Fig. S1; Table S1. [file aac.00784-25-s0001.docx]

Phase 1, Randomized, Double-Blind, Placebo-Controlled, Ascending Single- and Multiple-Dose Study of the Safety, Tolerability, and Pharmacokinetics of intravenous Xeruborbactam (QPX7728) in Healthy Adult Subjects

**SUPPLEMENTARY MATERIAL**

**Dosing schemes**

| Six SAD cohorts were originally planned. However, cohorts 3 and 5 were not conducted. Both were doses within the testing range and so the max and min doses were still performed. | | | | |
| --- | --- | --- | --- | --- |
| - SAD cohort 1: | 250 mg xeruborbactam |  |  | |
| - SAD cohort 2: | 500 mg xeruborbactam |  |  | |
| - SAD cohort 3: | 750 mg xeruborbactam | not conducted |  | |
| - SAD cohort 4: | 1000 mg xeruborbactam |  |  | |
| - SAD cohort 5: | 1500 mg xeruborbactam | not conducted |  | |
| - SAD cohort 6: | 2000 mg xeruborbactam |  |  | |
| On Day 1, subjects received a single IV dose of xeruborbactam or placebo as a 3-h infusion. | | | | |
| - MAD cohort 7 | 1000 mg IV xeruborbactam (loading dose) / 500 mg IV xeruborbactam | | |  |
| - MAD cohort 8 | 500 mg IV xeruborbactam (loading dose) / 250 mg IV xeruborbactam | | |  |
| For cohorts 7–8, on Day 1, subjects received a single dose of xeruborbactam or placebo. On Day 6, subjects received a loading dose of xeruborbactam, or placebo, followed by multiple doses every 8 h with the last dose on Day 12. Doses were administered as a 3-h IV infusion. | | | | |
| - MAD cohort 9 | 1000 mg IV xeruborbactam | | | |
| In cohort 9, subjects received IV doses of xeruborbactam or placebo as a 30-min IV infusion every 24 h on Days 1–7, with the last dose in the morning of Day 7. | | | | |

**Exclusion criteria**

History or presence of cardiovascular, pulmonary, hepatic, renal, haematological, gastrointestinal, endocrine, immunologic, dermatologic, neurological, or psychiatric disease; positive testis for HIV, hepatitis B surface antigen, or hepatitis C antibodies; history of substance abuse; use of prescription medication (except hormone replacement therapy for females) within 14 days prior to Day 1; use of over-the-counter medication, herbal products, probiotics, or vitamins within 7 days prior to Day 1; use of antacids, H2 receptor blockers, or proton pump inhibitors within 3 days prior to Day 1; documented hypersensitivity or anaphylaxis to any medication, including beta-lactam antibiotics; blood donation or significant blood loss within 56 days prior to Day 1; plasma donation within 7 days prior to Day 1; participation in another investigational clinical trial within 30 days prior to Day 1 or within 5 half-lives of the previous investigational drug (whichever is longer); significant acute illness within 30 days prior to Day 1; calculated Cockcroft-Gault creatinine clearance <80 mL/min at screening or Day 1; and clinically significant laboratory abnormalities at screening or Day 1.

**Data collection**Data collected before and during the study included age, sex, height, weight, BMI, vital signs, electrocardiogram monitoring, and clinical laboratory tests, including haematology, coagulation, serum chemistry, urinalysis, and serology.

(A)

(B)

**Supplementary Figure S1**. Protein binding. Relationship between plasma protein binding and total concentration in plasma (A) and between unbound concentration in ultrafiltrate and total concentration in plasma (B) for xeruborbactam in the range of 250–2000 mg. Vertical dashed lines indicate the mean total plasma concentrations for each dose group.

**Supplementary Table S1**. Blood and urine pharmacokinetic sampling schemes

| **Cohort** | **Day** | **Specimen** | **Sampling time** |
| --- | --- | --- | --- |
| SAD cohorts (3-h IV infusion) | Day 1 | Blood | Pre-dose, 1.5 (mid-infusion), 3 (end of infusion), 4, 5, 6, 7, 8, 12, and 24 h post-dose. |
|  |  | Urine | 0–4, 4–8, 8–12, and 12–24 h post-dose. |
| MAD cohort 7 (3-h IV infusion) | Day 1 | Blood | Pre-dose, 1.5, 3, 4, 5, 6, 7, 8, 12, 24, 36, 48, 60, 72, 96, and 120 h post-dose (pre-dose on Day 6). |
|  |  | Urine | 0–4, 4–8, 8–12, 12–24, 24–36, 36–48, 48–72, 72–96, and 96–120 h post-dose. |
|  | Day 6* | Blood | Pre-dose, 1.5, 3, 4, 5, 6, 7, and 8 h post-dose. |
|  | Day 12 | Blood | Pre-dose, 1.5, 3, 4, 5, 6, 7, 8, 12, 24, 36, 48, 60, 72, 96, and 120 h post-dose. |
|  |  | Urine | 0–4, 4–8, 8–12, 12–24, 24–36, 36–48, 48–72, 72–96, and 96–120 h post-dose. |
| MAD cohort 8 (3-h IV infusion) | Day 1 | Blood | Pre-dose, 1.5, 3, 4, 6, 8, 12, 24, 36, 48, 60, 72, 96, and 120 h post-dose (pre-dose on Day 6). |
|  |  | Urine | 0–4, 4–8, 8–12, 12–24, 24–36, 36–48, 48–72, 72–96, and 96–120 h post-dose. |
|  | Day 6* | Blood | Pre-dose, 1.5, 3, 4, 6, and 8 h post-dose. |
|  | Day 12 | Blood | Pre-dose, 1.5, 3, 4, 6, 8, 12, 24, 36, 48, 60, 72, 96, and 120 h post-dose. |
|  |  | Urine | 0–4, 4–8, 8–12, 12–24, 24–36, 36–48, 48–72, 72–96, and 96–120 h post-dose. |
| MAD cohort 9 (30-min IV infusion) | Day 1 | Blood | Pre-dose, 0.5, 1, 2, 4, 6, 8, 12, and 24 h post-dose. |
|  |  | Urine | 0–4, 4–8, 8–12, and 12–24 h post-dose. |
|  | Day 7 | Blood | Pre-dose, 0.5, 1, 2, 4, 6, 8, 12, 24, 36, 48, 60, 72, 96, and 120 h post-dose. |
|  |  | Urine | 0–4, 4–8, 8–12, 12–24, 24–36, 36–48, 48–72, 72–96, and 96–120 h post-dose. |
| *MAD cohorts loading dose. Post-dose: after the start of the infusion. | | | |

For PK blood samples, a 10-min deviation window was permitted for nominal times 0–4 h, 20-min for 8–24 h, and 30-min for times >24 h. After collection, blood was centrifuged, and plasma was collected. Plasma was diluted 1:1 with 3-(-N-morpholino) propanesulfonic acid (MOPS) buffer, thoroughly mixed, and frozen. For PK urine samples, a 20-min window was permitted for nominal times 0–24 h, and 60-min for times >24 h. Urine was refrigerated during collection intervals. At the end of each interval, total urine volume was weighted, recorded, and frozen.
